# IL-15–PI3K–AKT–mTOR: A Critical Pathway in the Life Journey of Natural Killer Cells

**DOI:** 10.3389/fimmu.2015.00355

**Published:** 2015-07-20

**Authors:** Alaa Kassim Ali, Neethi Nandagopal, Seung-Hwan Lee

**Affiliations:** ^1^Department of Biochemistry, Microbiology and Immunology, Faculty of Medicine, University of Ottawa, Ottawa, ON, Canada

**Keywords:** natural killer cells, IL-15, PI3K, AKT, mTOR, proliferation, effector functions, virus infection

## Abstract

Among numerous cytokines modulating natural killer (NK) cell function, interleukin 15 (IL-15) exerts a broad range of effect from development and homeostasis, to activation of mature NK cells during infection. Its significance is further highlighted by clinical trials in which IL-15 is being used to boost the proliferation and anti-tumor response of NK cells. Among the signal transduction pathways triggered by the engagement of IL-15 receptor with its ligand, the PI3K–AKT–mTOR pathway seems to be critical for the IL-15-mediated activation of NK cells, therefore being responsible for efficient anti-viral and anti-tumor responses. This review provides an overview of the role of IL-15 at multiple stages of NK cell life journey. Understanding the pathway by which IL-15 conveys critical signals for the generation of NK cells with efficient effector functions, in combination with established protocols for NK cell expansion *ex vivo*, will undoubtedly open new avenues for therapeutic applications for immunomodulation against infections and cancers.

## Introduction

Natural killer (NK) cells play critical roles in innate immune responses mainly against intracellular viruses and tumors (1–3). The term NK was originally derived from the observation that they are poised to kill target cells without prior sensitization or further differentiation (4, 5). Even though recognition receptors that target infected cells are germline-encoded with restricted recognition compared to T and B cell receptors, the mechanism of NK cell cytotoxicity against virus-infected cells is similar to that of CD8^+^ cytotoxic T lymphocytes (CTL). NK cells induce perforin/granzyme-dependent apoptosis in target cells and thereby eliminate reservoirs of virus replication (2, 3, 6). For this, NK cells express an array of inhibitory and activating receptors, which receive multiple stimuli from virus-infected cells. The balance between signals that are generated from activating receptors and inhibitory receptors mainly determines the immediate cytotoxic activation. NK cell activation also induces cytokine secretion such as IFN-γ and TNF-α. These cytokines enhance the phagocytic function of macrophages and their antimicrobial activity, and augment the adaptive immune response via up-regulation of antigen presentation by antigen presenting cells such as dendritic cells (DCs) (2, 7).

In addition to signals from both NK cell activating and inhibitory receptors, signals from cytokine receptors on NK cells greatly modulate the development and activation of NK cells. Even though cytokines can exhibit pleiotropic effects depending on the responding cell’s condition, IL-2, -12, -15, -18, and -21 are regarded as being stimulators for NK cell function (8, 9) whereas TGF-β and IL-10 are known as negative regulators (9). Notably, IL-15 is the most potent among them by orchestrating the entire life of NK cells and holds a great potential for immunotherapy in cancer and infectious research. Here, we will provide an overview of our knowledge regarding the PI3K–AKT–mTOR pathway as a major IL-15-mediated pathway driving the development, proliferation, and effector functions of NK cells.

KEY CONCEPT 1. mTOR Pathway The mammalian target of rapamycin (mTOR) is a serine/threonine protein kinase that functions as a sensor of environmental nutrient availability, and translates the information into cellular signals that regulate ribosomal biogenesis, protein translation, cell growth, and cell proliferation.

## Interleukin-15

IL-15 drew much attention due to its similarity to IL-2 in its cytokine receptor biology. IL-15R is a heterotrimeric receptor consisting of a unique α chain, a shared β subunit with IL-2 (CD122) and a common γ subunit (CD132) shared with several cytokines (10), implying similar biological activities of IL-2 and IL-15. IL-2 was the first cytokine used in clinical cancer trials due to its great ability to expand NK cells and stimulate NK cell functions (11). However, severe toxicity associated with the administration of IL-2 limited its application. Recently, the clinical interest of using IL-15 was further strengthened because IL-2 administration also expanded regulatory T cells, which suppress the anti-tumor response of NK cells and effector T cells (12–14). On the other hand, IL-15 therapy resulted in low toxicity with expansion of NK cells without expansion of Tregs *in vivo*, in a study of non-human primates (15).

KEY CONCEPT 2. IL-15 IL-15 is a key cytokine exhibiting a pleiotropic effect on the development, proliferation and activation of natural killer cells, as well as the proliferation and activation in CD8^+^ T-cell, therefore being considered as one of most promising molecules for anti-cancer immune therapy.

Studies have shown that IL-15^-/-^ mice exhibit a selective loss of memory phenotype CD8^+^ T cells, NKT cells, and NK cells (10, 16, 17). A unique aspect of IL-15 is that it employs trans-presentation in which IL-15-producing cells present the cytokine in the context of IL-15 receptor α (IL-15Rα) chain on the cell surface. A pivotal role of dendritic cell (DC)-expressed IL-15Rα for trans-presenting IL-15 to NK cells has been demonstrated (18, 19). Engagement of IL-15R on NK cells causes the auto-phosphorylation and activation of Janus Kinases (JAK1 and JAK3), which induces at least three parallel signaling cascades: Ras–Raf–MAPK, signal transduction and activation of transcription (STAT) 5 and PI3K–AKT–mTOR pathways (10, 20). The indispensable role of IL-15–STAT5 pathway for NK cell development and homeostasis was also exemplified in mice having defects in the signaling pathway in which the number of mature NK cells was dramatically reduced (21–23). Data from *Stat5*-deficient and NK cell-specific *Stat5*-deficient mice showed that NK cells are absent in peripheral lymphoid organs, suggesting a crucial role of the IL-15–STAT5 pathway in NK cell development (21–23). In addition, similarly to STAT5 knock-out mice, a severe reduction in NK cell numbers was found in a patient carrying the *STAT5b* mutation (24).

## PI3K–AKT–mTOR Pathway

In addition to the IL-15–STAT5 pathway, accumulating data pointed out that the PI3K–AKT–mTOR pathway is essential for modulating the development, differentiation, and activation of immune cells including NK cells. Signaling triggered by cytokines employing the JAK–STAT pathway generally stimulates the PI3K/AKT signaling pathway in immune cells (25). PI3K, phosphatidylinositol 3-kinase, is conserved in all mammalian cells and is known to control diverse processes including cell proliferation, survival, differentiation, activation of effector functions, and metabolism (26, 27). Among three classes (I, II, and III), the class I PI3Ks, which are heterodimeric enzymes consisting of a regulatory subunit (p85) and a catalytic subunit (p110), predominately regulate downstream signals emanating from cytokine receptor activation. Upon cytokines binding to their receptors, receptor tyrosine kinases activate PI3K, which generates phosphatidylinositol trisphosphate (PIP3) from plasma membrane-associated phosphatidylinositol bisphosphate (PIP2). PIP3 has an affinity for pleckstrin homology (PH) domain-containing molecules such as AKT and phosphoinositide-dependent protein kinase (PDK1) on the inner leaflet of the plasma membrane. At the plasma membrane, the interaction between the PH domain of AKT and PIP3 induces important conformational changes in AKT, which allow subsequent modifications of AKT at threonine 308 by PDK1. mTORC2 also can phosphorylate AKT at serine 473 for further activation (28).

Activated AKT phosphorylates crucial targets and contributes to cell survival by inhibiting pro-apoptotic members of the Bcl-2 family. One of the important downstream effectors for the PI3K/AKT signaling is mTOR, which is a serine/threonine protein kinase required for the translation of proteins that promote cell survival and proliferation. mTOR exists as two complexes, mTORC1 and mTORC2. Even though mTORC2 can activate mTORC1 by AKT phosphorylation, a metabolic reprograming which supports effector T cell proliferation and functions has been mainly investigated in the context of mTORC1 complex. mTORC1 is negatively regulated by a heterodimeric protein complex called tuberous sclerosis complex (TSC) 1 and 2. The TSC inhibits mTORC1 by suppressing the conversion of Rheb-GDP to Rheb-GTP, a small GTPase, required for mTORC1 activation. PI3K–AKT signaling results in the phosphorylation and inactivation of TSC2, which increases Rheb-GTP and mTORC1 kinase activity (29–32). mTORC1 promotes the translation machinery through the phosphorylation of the translation-initiation factor eIF4E-binding protein (4EBP1), and the S6 ribosomal kinase (S6K). Upon phosphorylation, the translation repressor protein 4EBP1 is dissociated from eIF4E, leading to the subsequent formation of the translation initiation complex. S6K directly phosphorylates several proteins implicated in protein translation including eukaryotic initiation factors and ribosomal protein S6 (33). In addition, mTORC1 increases the rate of glycolysis by inducing the expression of HIF-1α and c-Myc and nutrient transporters (30).

## PI3K–AKT–mTOR Pathway for NK Cell Development

**Figure 1:**
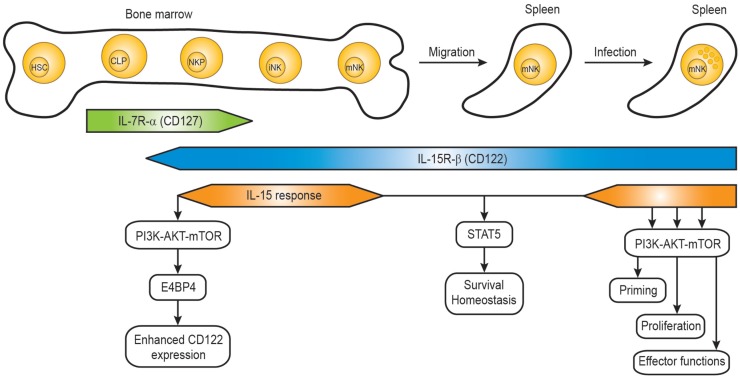
**IL-15 response during natural killer cell development**. The developmental stages of mouse NK cells in the bone marrow and periphery are shown, together with the IL-15R expression and IL-15 response. HSC, hematopoietic stem cell; CLP, common lymphoid progenitor; NKP, NK precursor; iNK, immature NK cell; mNK, mature NK cell.

Mature NK cells are differentiated from common lymphoid progenitors (CLPs). Even though NK cells can develop in extra-medullary sites such as the thymus and liver, the developmental program from CLPs to mature NK cells mainly occurs in the bone marrow (34, 35). CLPs differentiate into NK cell progenitors which are defined as Lin- NK1.1- CD122^+^ cells (36) and the acquisition of IL-15R-β chain (CD122) is a critical step allowing the progenitor cells to become responsive to IL-15 in the bone marrow compartment (Figure 1). Interestingly, NK cell progenitors display high proliferative potentials which are dependent on IL-15. Several studies from immune cell-specific deficient mice or *in vitro* NK cell differentiation identified factors responsible for the IL-15-mediated development process (35, 37).

Several identified factors are required for the acquisition and maintenance of CD122 on NK cell progenitors. The T-box transcription factor Eomes (also known as Eomesodermin) was shown to bind the CD122 promoter region, and the expression of CD122 on NK cells and memory CD8^+^ T cells from Eomes-deficient mice was significantly lower resulting in reduced responsiveness to IL-15 (38). The basic leucine zipper transcription factor E4BP4 (also known as Nfil3) seems to function upstream of Eomes, so that E4BP4 deficiency caused severe defects in NK cell development (39, 40). A recent paper demonstrated that PDK1, a kinase downstream of PI3K and upstream of mTOR, functions as a critical component in the positive feedback loop (41). Rescue of the defect of PDK1 by ectopic expression of E4BP4 or Eomes suggests that PDK1 signaling is critical for NK cell development via induction of E4BP4 and Eomes. Therefore, the IL-15 positive feedback loop might involve IL-15–PDK1–mTOR–E4BP4-Eomes–high levels of CD122-enhanced IL-15 signaling (Figure 1). The role of the metabolic checkpoint kinase mTOR on controlling NK cell proliferation in the bone marrow was also demonstrated in NK cell-specific mTOR-deficient mice (*Ncr1-Cre × Mtor^lox^*) (42). Consistently, a severe defect in early NK cell development was also observed in mice deficient in both the p110γ and p110δ catalytic subunits of PI3K (43), suggesting that PDK1 is a kinase linking IL-15–PI3K with the activation of AKT by phosphorylation.

## PI3K–AKT–mTOR Pathway for NK Priming Effect

The priming effect is based on the fact that exposure of IL-15 sensitizes NK cells to secondary stimuli, thereby resulting in a heightened response. It is a physiologically relevant process because NK cells are recruited to lymph nodes where they are first activated by trans-presentation of IL-15 by IL-15Rα expressed on DCs (44) during inflammation and stimulated by subsequent cytokines or activating ligands. A well-known “priming” is exemplified by an exaggerated IFN-γ response when NK cells are co-stimulated with IL-12, IL-18, and IL-15 *ex vivo* (44–46). In our previous work, we demonstrate that the priming effect by IL-15 can be extended to stimulation by most cytokines employing the JAK–STAT pathway and ligands of NK cell activation receptors (47). When the priming effect is evaluated by measuring the phosphorylation of respective STATs, the response to type I IFN, IL-21, IL-12, IL-2, and IL-4 stimulation in NK cells primed by IL-15 was found greatly enhanced compared to naïve NK cells. In addition, the IFN-γ expression in primed NK cells after stimulation through Ly49H activation receptor was increased.

KEY CONCEPT 3. Priming Effect The priming effect of cytokines refers to a phenomenon in which one cytokine can increase the sensitivity of cells to stimulation with other cytokines, resulting in an exaggerated response.

Several mechanisms have been proposed to explain the priming effect. Stimulation with IL-12 or IL-15 can induce the up-regulation of IL-18R transcript in human naive CD56^+^ NK cells, therefore enhancing the response to subsequent IL-18 exposure and IFN-γ production (45, 48). In our study, PI3K–AKT–mTOR pathway was identified as a principal pathway inducing the priming effect. Blocking of individual components of the pathway using cell-permeable reversible inhibitors severely abrogates the IL-15-mediated priming effects, as tested for, at least, IL-12-induced phosphorylation of STAT4, IL-21-induced phosphorylation of STAT3, and Ly49H-induced IFN-γ production. One caveat of using pharmacological inhibitors is that some inhibitors could modulate off-targets. In particular, Ly294002, a common PI3K inhibitor, has been known to induce non-specific effects, including the direct inhibition of mTOR’s catalytic function (49). Therefore, data obtained from experiments using Ly294002 should be taken cautiously. Nonetheless, inhibitors used for blocking AKT and mTOR in our study were generally regarded as highly specific. It is noteworthy that the requirement of mTOR for JAK–STAT activation might be extended to T cell regulation. Similar to the diminished levels of phosphorylated STATs in rapamycin-treated IL-15-primed NK cells, mTOR-deficient CD4 T cells fail to differentiate into Th1, Th2, and Th17 cells and this defect was largely due to impaired phosphorylation of respective STAT molecules required for each lineage differentiation (50). Mechanisms by which the PI3K–AKT–mTOR pathway crosstalks with the JAK–STAT pathway for inducing the priming effect are still not clear. It would be interesting to investigate the role of PI3K–AKT–mTOR pathway in the regulatory pathways of general JAK–STAT signaling such as suppressor of cytokine signaling (SOCS), protein inhibitors of activated STAT (PIAS), and protein tyrosine phosphatases (PTPs) (51).

Recently, a new form of priming using IL-15, IL-12, and IL-18 was suggested to generate memory-like NK cells which show enhanced effector functions upon restimulation following a 3-week resting period (52). This memory-like phenotype was found in proliferating NK cells. Due to the long-lasting and enhanced effector phenotype, studies that are trying to use adoptive transfer of the memory-like NK cells pre-activated with IL-12/-15/-18 for cancer immunotherapy (8, 53, 54) are being carried out, and one group showed that adoptive transfer of those pre-activated NK cells greatly increases the numbers and effector functions of NK cells, resulting in pronounced tumor regression in a mouse model of established tumor (53). One of the mechanisms responsible for this prolonged and enhanced tumor surveillance was mediated through IL-2Rα chain (CD25) up-regulation induced by IL-12 and IL-18 stimulation, thereby allowing NK cells to be sustained under the low physiological levels of IL-2 (53, 55). Nonetheless, whether PI3K–AKT–mTOR pathway is required to induce memory-like NK cells has not been tested.

## PI3K–AKT–mTOR Pathway for NK Cell Proliferation During Virus Infection

During their differentiation in the bone marrow, NK cells hold a great potential of proliferation. However, NK cells become gradually quiescent as they fully mature (Figure 1) (56, 57). Ample data support that IL-15–JAK1/3-STAT5 is required for the homeostasis and survival of peripheral mature NK cells (10, 58, 59). Since IL-15 is essential for NK cell survival, cells deprived of IL-15 undergo considerable death. When adoptively transferred to IL-15^-/-^ mice, mature splenic NK cells failed to be maintained, mainly due to the inability to sustain sufficient levels of the anti-apoptotic Bcl-2 expression *in vivo* (60, 61). Moreover, *in vivo* antibody blocking of IL-15R signaling also resulted in a severe loss of splenic NK cells within a week. Consistent with the previously identified indispensable role of IL-15-JAK1/3–STAT5, blocking of JAK and STAT5 pathways resulted in significant cell death, similar to when NK cells are deprived of IL-15. Notably, NK cells treated with inhibitors for the PI3K–AKT–mTOR were still able to maintain their viability similar to controls (cells without any inhibitor treatment) in the presence of IL-15, suggesting that the pathway is dispensable to support the survival of mature NK cells (47). The dispensable role of mTOR for survival was further supported in the study showing intact prosurvival signals provided by IL-15 in mTOR-deficient NK cells (21, 42).

**Figure 2:**
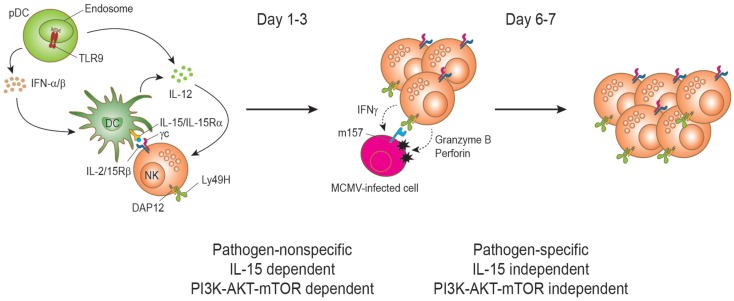
**Natural killer cell proliferation during murine cytomegalovirus infection**. During MCMV infection, two stages of NK cell proliferation showing pathogen non-specific and specific responses have been proposed. IL-15 drives NK cell proliferation during the early phase of MCMV infection. Upon recognition of CpG motifs from MCMV viral DNA by TLR9, plasmacytoid dendritic cells (pDCs) secrete type I interferons (IFNα/β) and interleukin-12 (IL-12) cytokines. DC-derived IL-12 stimulates NK cells to produce IFN-γ. IFNα/β is transiently produced and reaches a peak level at day 1.5 post-infection and the production is important to induce the expression of IL-15. IL-15 is trans-presented by DCs to NK cells to induce proliferation and enhanced effector functions of NK cells. Activated NK cells can induce perforin/granzyme-mediated apoptosis of MCMV-infected cells by recognizing the viral m157 protein on the cell surface. This direct recognition depends on the activating receptor Ly49H. Proliferation at this stage is dependent on the PI3K–AKT–mTOR pathway. The interaction between Ly49H and the MCMV-encoded protein m157 drives the proliferation and expansion of Ly49H^+^ NK cells on days 6–7 post infection. Notably, this proliferation can occur in IL-15- and IL-15Rα-deficient mice during MCMV infection and is independent of the PI3K–AKT–mTOR pathway.

Mature peripheral NK cells can rapidly and intensively proliferate during viral infections. The evidence for NK cells proliferation during virus infections was first recognized by Biron and colleagues three decades ago when they observed transient NK cell blastogenesis characterized by increased cell mass and proliferation at the onset of lymphocytic choriomeningitis virus (LCMV) and murine cytomegalovirus (MCMV) infections (62–64). Early on during the course of MCMV infection, blastogenesis and proliferation of NK cell is dependent on IL-15 (65, 66) (Figure 2). The IL-15 trans-presentation and the resulting activation of NK cells are dependent on type I IFN mainly induced by TLR recognition of viral PAMP (44). Our study pointed out that the PI3K–AKT–mTOR pathway is essential for the proliferation of mature NK cells upon IL-15 stimulation (47). *Ex vivo* IL-15 stimulation of splenic NK cell in the presence of inhibitors for the pathway resulted in a dramatic reduction of proliferation. The defect was consistently observed in a broad range of IL-15 concentrations. To test the role of the pathway *in vivo*, we decided to evaluate NK cell proliferation in mice treated with rapamycin to block mTOR pathway during MCMV infection, where NK cell proliferation at early infection is well reported. During MCMV infection, NK cells show a peak proliferation response at days 2.5–3 post infection. In line with *ex vivo* data, proliferation of NK cells as measured by BrdU incorporation was severely reduced in rapamycin-treated mice during MCMV infection. This mTOR-dependent NK cell proliferation has been reproduced in an independent study showing that early proliferation during MCMV infection was greatly impaired in NK cell-specific mTOR-deficient mice (42).

Yokoyama and colleagues proposed two stages of NK cell proliferation consisting of pathogen-non-specific and -specific proliferation during MCMV infection (57) (Figure 2). Following the early IL-15-induced NK cell proliferation (first stage), the proliferation can be extended to a later stage of preferential proliferation of NK cells expressing the specific activating receptor that recognizes a viral ligand on the infected cells (second stage). The main example is the Ly49H-activating receptor that recognizes the MCMV-encoded m157 glycoprotein on MCMV-infected cells (67–71). This recognition induces perforin/granzyme-mediated killing of the infected cells and confers NK cell-mediated resistance to MCMV (2). During MCMV infection, Ly49H^+^ NK cells display preferential proliferation and the expansion is blocked by α-Ly49H antibody treatment, indicating that a stimulatory signal through the Ly49H is responsible for the proliferation. This pathogen-specific NK cell proliferation and expansion is comparable to that of antigen-specific T cells during virus infection and shows a peak response on days 6 and 7 post infection (72). Analogous NK cell expansion has also been found in other virus infections such as hantavirus, HCMV, and HIV (73–75). Notably, NK cells in NK cell-specific mTOR-deficient mice displayed substantial Ly49H-dependent proliferation at day 6.5, even though less proliferation was observed compared to mTOR-sufficient NK cells (42). Therefore, mTOR pathway is critical in IL-15-driven proliferation, but has a minimal role on activating receptor-driven proliferation. Such IL-15-independent and Ly49H-mediated NK cell proliferation has previously been reported (76).

## PI3K–AKT–mTOR Pathway for NK Cell Effector Function During Virus Infection

Natural killer cells were originally regarded as “ready to kill” cells that can immediately destroy virus-infected or transformed cells (5, 77). However, unlike human NK cells, naïve mouse NK cells derived from laboratory strains of mice housed in a specific pathogen-free environment are devoid of perforin and granzyme B cytotoxic granules and exhibit minimal effector functions (44, 78, 79). A report demonstrated that IL-15 can induce rapid translation of pre-existing perforin and granzyme B mRNAs in NK cells in order to be fully equipped for action (78). Accordingly, splenic NK cells are induced to express high levels of granzyme B upon stimulation with IL-15 for 24 h. Notably, the granzyme B induction was abrogated in the presence of inhibitors blocking the PI3K–AKT–mTORC1 pathway (47). Recent reports have demonstrated that mTORC1 plays a major role in IL-15-induced NK cell activation. mTORC1 activity is greatly increased in mice treated with poly IC (a mimic of virus infection and inducer of IL-15 expression) or infected with MCMV, as exemplified by the drastic increase in the mTORC1-mediated phosphorylation of S6 ribosomal protein, a downstream target (42, 47, 80). Both pharmacological inhibition and genetic ablation demonstrated that mTORC1 is a non-redundant metabolic regulator for NK cell activation, dictating proliferation, and effector functions upon poly IC treatment (42, 80). Ablation of mTORC1 signaling reduced blastogenesis, and inhibited the expression of effector molecules such as IFN-γ and granzyme B.

To translate the importance of PI3K–mTOR pathway for NK cell functions during virus infection, we treated mice with rapamycin to block mTOR kinase activity downstream of PI3K (30, 81). The treatment of rapamycin on NK cell functions during MCMV infection also resulted in severe defects in IFN-γ and granzyme B productions in addition to defects in proliferation. Similar impairments were also observed in NK cells treated *ex vivo* with inhibitors for activating kinases upstream of mTORC1 such as PI3K and AKT (47). It is well established that granzymes and IFN-γ are effector molecules required for the efficient anti-viral activity of NK cells (82–85). Therefore, *in vivo* treatment with the mTORC1 inhibitor rapamycin during MCMV infection abrogated NK cell cytotoxicity toward YAC-1 tumor cells and resulted in elevated viral loads in the infected organs (47). This was accompanied by defective NK cell effector functions, thereby coupling this important metabolic sensor to NK cell anti-viral responses. Taken together, the results demonstrated the central role of PI3K–AKT–mTORC1 pathway in IL-15-induced NK cell activation of effector functions during virus infection.

During virus infection, activated NK cells must increase their metabolic uptake to support blastogenesis and the accelerated cell cycles. It includes elevated production of biomolecules such as nucleic acids, proteins, and lipids accompanied by the increased uptake of nutrients such as glucose and increased oxygen consumption. The major role of IL-15 in the metabolic changes in NK cells during virus infection was supported by the observation that up-regulated genes associated with metabolic pathways are largely shared in microarray data of NK cells from mice infected with MCMV *in vivo* or of NK cell treated with IL-15 *in vitro* (42). NK cells from both conditions induce increased glucose uptake and increased expression of the nutrient receptors CD71 (transferrin receptor) and CD98 (a component of the l-amino acid transporter). So how does IL-15 fulfill such heightened metabolic demands in NK cells during virus infection? Among the pathways activated upon IL-15 engagement, it seems that the IL-15–PI3K–mTOR pathway is critical for the metabolic reprograming. Whereas mTOR senses environmental nutrient availability and translates the information into cellular responses (50, 86, 87), it has become evident that mTOR-regulated cellular metabolism plays a fundamental role in dictating immune cell differentiation and function (88, 89). mTOR-regulated glycolysis is also linked to the differentiation of activated CD4^+^ T cells into anti-microbial Th1 and Th17 cells (50, 90, 91). In TCR-stimulated CD8^+^ T cells, the PI3K–mTORC1 pathway was also suggested to activate lipid synthesis for membrane biosynthesis during blastogenesis (92).

## Concluding Remarks

In this review, we have discussed how the IL-15–PI3K–mTOR signaling pathway controls distinct stages of NK cell life cycle. Accumulated evidence demonstrated that the signaling axis of IL-15–PI3K–mTOR in NK cells is important for their development, cellular proliferation, responsiveness to cytokine stimulations, and cytotoxic functions. mTOR is a key regulator of cell growth and metabolism and also provides a link between the metabolism and effector functions of immune cells. IL-2/IL-15 share the receptor subunits IL-2/IL-15R-β and -γ chains and both are being widely used for *ex vivo* expansion of NK cells in immunotherapy. Their promising therapeutic capacity for a variety of human malignancies has stimulated an interest in using NK cells for anti-cancer treatments (93, 94). Recently, IL-15 rather than IL-2 is the preferred cytokine for expanding NK cells *in vivo* due to the minimal concern about toxicity and expansion of Treg (95). Therefore, understanding the molecular mechanisms by which IL-15 primes and activates NK cells will allow the manipulation of IL-15 signaling for improving NK cell-based therapeutic strategies against cancers and infectious diseases.

Interestingly, even though the IL-15–PI3K–AKT–mTOR pathway regulates multiple processes for NK cell biology from development in the bone marrow to activation in the periphery, some factors such as E4BP4 and PDK1 required for IL-15-mediated development stages of NK cells are dispensable for differentiation, survival, or effector function of mature NK cells (39, 95, 96). Therefore, it suggests that additional signaling modules might need to incorporate with the common IL-15–PI3K–AKT–mTOR pathway in order to induce stage-specific processes in NK cells.

The NK cell proliferation, cytokine production, and cytotoxicity are dependent on the metabolic induction of mTOR pathway during virus infection (42, 47, 80). Therefore, understanding the metabolic program utilized by NK cells will guide the development of optimal NK cell-mediated therapeutic interventions. Such metabolic modulations on immune cells have been used to induce enhanced CD8^+^ T cell memory responses. For instance, treatment of rapamycin reduces glycolysis and promotes the lipid metabolic program (50, 97), which is favorable for the generation of memory CD8^+^ T cells (98–100); therefore suggesting its potential application to enhance vaccine efficacy against viruses. The study of the metabolic regulation of NK cells is still in its infancy compared to works established in T cells. Our current knowledge has been mainly limited to cell culture experiments *in vitro*; therefore translation of these findings for *in vivo* immunotherapy warrants caution and careful interpretation. Without any doubt, modulating the immune responses by manipulating cellular metabolic pathways may provide an innovative option for cancer immunotherapy.

## Conflict of Interest Statement

The authors declare that the research was conducted in the absence of any commercial or financial relationships that could be construed as a potential conflict of interest.
